# Survival analysis of patients with rheumatic MS after PBMV compared with MVS in a low-to-middle-income country

**DOI:** 10.1007/s12471-019-01315-x

**Published:** 2019-07-29

**Authors:** A. M. Ambari, B. Setianto, A. Santoso, B. Dwiputra, B. Radi, A. A. Alkatiri, A. B. Adji, E. Susilowati, F. Tulrahmi, M. J. M. Cramer, P. A. Doevendans

**Affiliations:** 1 Department of Cardiology Vascular Medicine, Faculty of Medicine, National Cardiovascular Center Harapan Kita, University of Indonesia, Jakarta, Indonesia; 2 Department of Thorax Cardio Vascular Surgery, Faculty of Medicine, National Cardiovascular Center Harapan Kita, University of Indonesia, Jakarta, Indonesia; 3 Division of Preventive and Rehabilitative, National Cardiovascular Centre Harapan Kita, Jakarta, Indonesia; 4 Department of Cardiology, University Medical Center Utrecht, Utrecht, The Netherlands

**Keywords:** Rheumatic mitral stenosis, Percutaneous balloon mitral valvulotomy, Mitral valve surgery, Survival analysis

## Abstract

**Introduction:**

Rheumatic mitral stenosis continues to be prevalent in developing countries, notably in endemic areas. Over the last few decades, percutaneous balloon mitral valvuloplasty (PBMV) has been established as a lower-cost alternative treatment for mitral stenosis (MS) in low-to-middle-income countries. PBMV has also been suggested to be an effective and safe alternative treatment modality. This study aims to analyse the survival of rheumatic MS patients treated with PBMV compared with those treated with mitral valve surgery (MVS).

**Methods:**

This study was a national, single-centre, longitudinal study using a survival analysis method in 329 consecutive patients suffering from rheumatic heart disease with severe MS who underwent PBMV compared with 142 consecutive patients with similar characteristics who underwent MVS between January 2011 and December 2016. Survival analysis and event-free duration were determined over a median follow-up of 24 months in the PBMV group and 27 months in the MVS group.

**Results:**

The results showed that of the 329 consecutive patients in the PBMV group, 61 patients (18.5) had an event (6 patients died and 55 patients were hospitalised), and of the 142 consecutive patients in the MVS group, 19 patients (13.4%) had an event (5 patients died, and 14 patients were hospitalised). The hazard ratio was 0.631 (95% confidence interval, 0.376–1.058; *P* = 0.081). Longer short-term survival was found in the MVS group but was not statistically significant. Event-free survival was significantly longer in the MVS group (*P* = 0.002), by 5 months.

**Conclusions:**

In this study, the efficacy and safety of PBMV was reconfirmed, as PBMV proved to be non-inferior to MVS in survival prognosis, but sustained event-free duration was significantly better in the MVS group than in the PBMV group.

## What’s new

- Percutaneous balloon mitral valvuloplasty is non-inferior compared with mitral valve surgery in prognostics for survival, but sustained event-free duration was significantly better in the mitral valve surgery group.
- Higher event-free survival was found in the mitral valve surgery group, but it was not statistically significant.
- Event-free duration was significantly 5 months longer in the mitral valve surgery group (*p* = 0.002).

## Introduction

Rheumatic heart disease (RHD) is a major burden in developing countries and causes most cardiovascular morbidity and mortality in children and young adults. The worldwide prevalence and annual incidence of RHD have been approximated to be >15 million cases and >280,000 cases per year, respectively. Recent studies in Asia have estimated an existing RHD burden of 10.8–15.9 million patients, accounting for 356,000–524,000 deaths per year [1]. RHD is a progressive and chronic condition caused by complement-mediated damage to the atrioventricular valves, frequently including mitral stenosis, that occurs as a result of the inflammatory response in rheumatic fever [2].

The safety and efficacy of percutaneous balloon mitral valvuloplasty (PBMV) and mitral valve surgery (MVS) have already been established. PBMV is a safe and well-tolerated intervention and is associated with short-term benefits [3]. The increasing burden to reduce health-related expenses makes it compulsory to provide an effective and safe yet economical intervention. Over the last few decades, PBMV has been established as a lower-cost alternative to MVS in low-to-middle-income countries [4–7]. This study was conducted to identify in-hospital survival and short-term survival of rheumatic mitral stenosis patients undergoing PBMV compared with those undergoing MVS.

## Methods

### Patient Selection

We conducted a single-centre, retrospective follow-up study. All 471 consecutive patients admitted with severe mitral stenosis with or without tricuspid valve repair or replacement between January 2011 and December 2016 were included. The study group comprised 329 patients who underwent PBMV and 142 patients who underwent MVS. Patients with congenital heart disease or those who received non-mitral valve surgery or coronary artery bypass grafting were excluded from this study. The data were acquired from medical records and local databases.

### Treatment Strategy

The definite treatment strategy for patients with severe MS, either PBMV or MVS, was determined during the pre-surgery conference attended by cardiologists and surgeons and was based on echocardiographic data, patient age, and comorbidities. Our institutional review board approved the retrospective analysis of the clinical data of these subjects.

Treatment indications followed the ESC guidelines for intervention in MS. PBMV was indicated in symptomatic patients with a valve area <1.5 cm^2^ if symptoms could not be explained by another cause, and if the anatomy was favourable, in symptomatic patients with a contraindication or a high risk for surgery, as well as in asymptomatic patients without unfavourable clinical or anatomical characteristics for PBMV with high thromboembolic risk and/or a high risk of haemodynamic decompensation. PBMV was contraindicated if the mitral valve area >1.5 cm^2^, if there was a presence of left atrial thrombus, if a patient had more than mild mitral regurgitation, in the case of severe or bi-commissural calcification, in the case of an absence of the commissural fusion, in patients with severe concomitant aortic valve disease or severe combined tricuspid stenosis, if a patient had regurgitation that required surgery, and in patients with concomitant coronary artery disease that required bypass surgery. Mitral valve surgery is indicated in symptomatic patients who are not suitable for PBMV [8]. The Wilkins score was calculated. A mitral valve with a score less than 8 indicated that the patient was a candidate for PBMV. In patients with a score ≥8, especially in those with more than moderate mitral regurgitation, surgical therapy was performed, except in patients with serious comorbidities [9].

### Treatment Technique

Percutaneous balloon mitral valvuloplasty procedures were performed by a percutaneous trans-septal anterograde approach and the Inoue balloon technique, according to the stepwise technique procedure, under echocardiographic guidance. The mitral valve area was calculated using the Gorlin formula [10]. MVS was performed with mitral valve replacement or mitral valve repair. Mitral valve replacement was conducted in 115 of 142 patients (81%), and mitral valve repair was conducted in 27 of 142 patients (19%).

### Measurement

The diagnosis of rheumatic mitral valve disease was made by echocardiography. Echocardiography was performed and analysed in the same centre. Rheumatic valve diseases were diagnosed using the World Heart Federation Criteria for Rheumatic Heart Disease. Comprehensive 2‑dimensional and colour Doppler echocardiographic evaluation was performed in all patients before PBMV or MVS. In addition to routine measurements of cardiac chamber dimensions and ejection fraction by the modified Simpson method, the mitral gradient and the peak pressure gradient of tricuspid regurgitation were calculated. The morphological features of the mitral valve were categorised as previously described, and the total echocardiographic score was obtained by adding the scores of each of the following individual morphological features: leaflet mobility, thickness, calcification, and subvalvular lesions. The mitral valve area was measured by direct planimetry to calculate the mitral gradient and the peak pressure gradient of tricuspid regurgitation.

### Follow-up

Follow-up data were collected from January until June 2017. The data were obtained either from medical records during patient visits to the outpatient clinic or during hospitalisation or by telephone interviews. The endpoints were defined as the clinical events of cardiovascular death and hospitalisation.

### Data Analysis

**Fig. 1:**
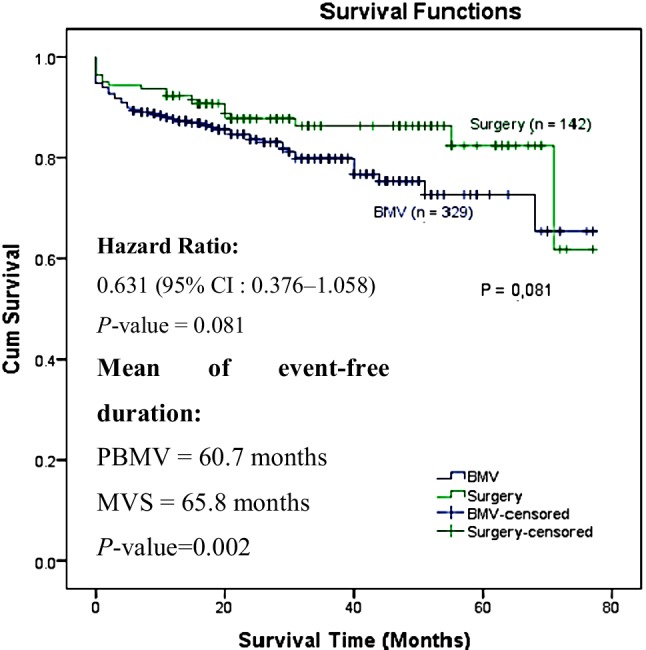
Cox regression & Kaplan-Meier curve analysis (*CI* confidence interval, *PBMV* percutaneous balloon mitral valvuloplasty, *MVS* mitral valve surgery, *BMV* balloon mitral valvuloplasty)

Categorical variables are presented as numbers and percentages and were compared by χ^2^ or Fisher’s exact test. Continuous variables and actuarial survival rates are expressed as the mean standard deviation and were compared by unpaired *t*-tests, except for follow-up duration, which is expressed as a median. The Cox proportional hazards model was used to determine whether the event-free survival differed significantly between PBMV and MVS patients after controlling for the differences in their pre-procedural risk profiles. Bivariate analysis was performed with a Cox model to determine the risk factors of an outcome. Variables were entered in a Cox multivariate model with a backward selection procedure and a significance level of *P* < 0.05 to prevent failure in identifying variables known to be important [11]. Two-way interactions were studied between these selected variables with a stratified log-rank test. The final Cox multivariate model was established by a backward selection of these variables with a significance level of *P* < 0.05. Cumulative survival curves were determined according to the Kaplan-Meier method (Fig. 1). The analysis was performed with SPSS.

## Results

### Baseline Characteristics and short-term Outcomes

This study involved a total of 471 patients, comprising 329 PBMV patients and 142 MVS patients. From 142 patients who underwent MVS, mitral valve repair was performed in 26 patients (18.3%) with mitral valve replacement in 116 patients (81.7%). The median follow-up was 24 months in the PBMV group and 27 months in the MVS group. The endpoints of the present study were the frequency of objective clinical events, including cardiovascular death and hospitalisation. The baseline characteristics of all patients are summarised in Tab. 1.

**Table 1:** Frequency distribution of patient characteristics in the PBMV and MVS groups

Variable	PBMV (*n* = 329)	MVS (*n* = 142)
Age, y	39.3 ± 1.6	41.2 ± 11.3
Male, *n* (%)	87 (26.4%)	57 (4.1%)
Smokers, *n* (%)	35 (10.6%)	28 (19.7%)
Diabetes mellitus, *n* (%)	6 (1.8%)	9 (6.3%)
Hypertension, *n* (%)	29 (8.8%)	13 (9.2%)
Dyslipidaemia, *n* (%)	3 (0.9%)	10 (7%)
Anticoagulant, *n* (%)	226 (68.7%)	142 (100%)
ACE inhibitor, *n* (%)	1 (0.3%)	86 (60.6%)
Beta blocker, *n* (%)	149 (45.3%)	68 (47.9%)
Digitalis, *n* (%)	146 (44.4%)	35 (24.6%)
Diuretic, *n* (%)	291 (88.4%)	87 (61.3%)
AF, *n* (%)	200 (60.8%)	83 (58.5%)
BMI, *n* (%):		
– Underweight	68 (20.7%)	46 (33.3%)
– Normal	136 (41.3%)	58 (42%)
– Overweight	57 (17.3%)	12 (8.7%)
– Obesity	68 (20.7%)	22 (15.9%)
Wilkins score	Median score = 7	Median score = 8

*PBMV* percutaneous balloon mitral valvuloplasty, *MVS* mitral valve surgery, *ACE* angiotensin-converting enzyme,* AF* atrial fibrillation, *BMI* body mass index

The youngest age of PBMV patients was 11 years, and the eldest was 72 years. In MVS patients, the mean age distribution of patients was 41.2 years (39.36–43.12), with a median age of 40 years and a standard deviation ±11.3. The youngest MVS patient was 16 years old, and the eldest was 67 years. In PBMV patients, we found mostly women with atrial fibrillation and normal BMI. More than 50% of patients used anticoagulant and diuretic medications.

From 329 BMV patients, we found that 61 (18.5%) patients had an event (6 patients died and 55 patients were hospitalised). From 142 surgery patients, 19 (13.4%) had an event (5 patients died and 14 patients were hospitalised). The frequency distribution of events is summarised in Tab. 2.

**Table 2:** Frequency distribution of events in the PBMV and MVS groups

Variable	PBMV (*n* = 329)	MVS (*n* = 142)
Event (mortality/morbidity), *n* (%)	61 (18.5%)	19 (13.4%)
Mortality, *n*	6	5
Rehospitalisation, *n*	55	14
– Congestive heart failure, *n*	49	13
– Repeat intervention, *n*	2	0
– Arrhythmia	3	0
– Cerebrovascular accident, *n*	1	1

*PBMV* percutaneous balloon mitral valvuloplasty, *MVS* mitral valve surgery

Bivariate analysis showed some predictors that could influence the study outcome. In PBMV and MVS patients, there was no association between these predictors and the observed events. The results of the bivariate analysis are summarised in Tab. 3. In the univariate Cox regression analysis, no variable affected patient survival following the PBMV or MVS procedure. Multivariate Cox regression was performed, showing that 3 variables became confounding factors in this study (Tab. 4). MVS patients were less likely to have events after adjusting for these variables (adjusted hazard ratio [HR] = 0.6).

**Table 3:** Bivariate analysis predictors of event outcomes

Variable	PBMV (*n* = 329)			MVS (*n* = 142)		
	*n*	HR (95% CI)	*P*-value	*n*	HR (95% CI)	*P*-value
Male	16 (18.4%)	1.0 (0.6–1.8)	0.977	8 (14%)	1.1 (0.4–2.7)	0.894
Age	Mean 39.3 ± 1.6	1.0 (0.9–1.0)	0.593	Mean 41.2 ± 11.3	1.0 (0.9–1.1)	0.194
Wilkins score	Median 7	1.1 (0.9–1.4)	0.485	Median 8	1.2 (0.7–1.1)	0.555
Smokers	6 (17.1%)	0.9 (0.4–2.3)	0.957	4 (14.3%)	1.0 (0.3–3.1)	0.979
DM	1 (16.7%)	0.8 (0.1–5.7)	0.812	0	–	0.456
Hypertension	8 (27.6%)	1.6 (0.8–3.4)	0.222	2 (15.4%)	1.3 (0.3–5.9)	0.692
Dyslipidaemia	1 (33.3%)	3.1 (0.4–22.4)	0.625	0	–	0.434
Anticoagulant	45 (19.9%)	1.3 (0.7–2.3)	0.404	19 (13.4%)	–	–
ACE inhibitor	1 (100%)	9.3 (1.3–67.7)	0.028	11 (12.8%)	0.9 (0.4–2.5)	0.954
Beta blocker	26 (17.4%)	0.9 (0.6–1.5)	0.743	10 (14.7%)	1.1 (0.5–2.8)	0.807
Digitalis	27 (18.5%)	0.9 (0.5–1.4)	0.588	3 (8.6%)	0.5 (0.1–1.7)	0.254
Diuretic	56 (19.2%)	1.6 (0.6–4.0)	0.313	10 (11.5%)	0.6 (0.2–1.4)	0.239
ECG (AF)	42 (21%)	1.5 (0.8–2.5)	0.173	10 (12%)	0.8 (0.3–1.9)	0.604
BMI:						
– Underweight	13 (19.1%)	1.0		5 (10.9%)	1.0	
– Normal	24 (17.6%)	0.9 (0.5–1.9)	0.944	6 (10.3%)	1.0 (0.3–3.3)	0.995
– Overweight	9 (15.8%)	0.8 (0.3–1.9)	0.643	1 (8.3%)	0.8 (0.1–6.9)	0.845
– Obesity	15 (22.1%)	1.1 (0.5–2.3)	0.791	6 (27.3%)	3.3 (0.9–10.9)	0.055

*PBMV* percutaneous balloon mitral valvuloplasty, *MVS* mitral valve surgery,* HR* hazard ratio*, CI* confidence interval*, DM* diabetes mellitus, *ACE* angiotensin-converting enzyme, *ECG* electrocardiography, *AF* atrial fibrillation, *BMI* body mass index

**Table 4:** Multivariate analysis predictors of event outcomes

Variable	HR	95% CI	*P*-value
Treatment	0.6	0.3–1.1	0.081
Wilkins score	1.1	0.9–1.4	0.303
Digitalis	0.7	0.4–1.1	1.124
Diuretic use	1.2	0.6–2.4	0.648

*HR* hazard ratio, *CI* confidence interval

## Discussion

The main finding of this study is that there were no significant differences in survival prognosis between groups. Short-term survival was similar in both groups, and the hazard ratio for the clinical events after MVS compared with PBMV was 0.631 (95% confidence interval [CI], 0.376–1.058; *P* = 0.081). Sustained life expectancy was found in the MVS group compared with the PBMV group. The event-free duration was significantly longer in the MVS group (*P* = 0.002), by 5 months.

The patient characteristics that were assessed in this study were sex, age, body mass index (BMI), smoking status, diabetes mellitus (DM), hypertension, dyslipidaemia, anticoagulant (warfarin) use, angiotensin-converting enzyme inhibitor use, beta blocker use, digitalis use, diuretic use and the presence of atrial fibrillation. These variables did not interfere with the survival results, because the bivariate analysis showed that none of them was significantly correlated with the outcome events. This result was in accordance with that of Song et al. (2010), who identified the long-term outcomes between PBMV and MVS in a total of 561 consecutive patients between January 1995 and December 2000, with a median follow-up of 109 months. The previous study showed that 20 patients (13%) who underwent MVS died, and 78 patients (19%) who underwent PBMV died. Based on the unadjusted survival results, both groups had the same event-free survival rate (HR = 1.51; 95% CI: 0.914–2.496; *P* = 0.1079). After the data were adjusted for age, left atrial anteroposterior diameter, and echo findings, the hazard ratio became 3.729 (95% CI: 1.962–7.082; *P*-value <0.001). The results led to the conclusion that patients who receive MVS have better longer-term survival rates than those who receive PBMV [12].

The results of research conducted by Chen et al. (2015) indicated that PBMV is a safe and effective procedure for RHD patients with MS and tricuspid regurgitation. This procedure can relieve symptoms, reduce the magnitude of tricuspid regurgitation and can improve the quality of life and patient prognosis, but the long-term effects need to be monitored. In this study, the tricuspid regurgitation area (TRA) increased when the mitral valve area (MVA) decreased, and there was a reverse relationship between the two (*r* = −0.8, *t* = 27.115, *P* < 0.01). In particular, tricuspid regurgitation contributes to increased morbidity and mortality despite surgical and percutaneous measures of mitral valve disease [13]. Kim et al. (2007) compared long-term outcomes after mitral valve replacement or repeated PBMV in patients with restenosis after a previous balloon valvotomy. In a survival analysis until 40 months postoperatively, both procedures had the same event-free survival, but 6 years and 9 years of follow-up showed that mitral valve replacement had significantly longer event-free survival than repeated PBMV [10]. Additionally, a study by Lee et al. (2015) aimed to determine the outcomes of mitral valve repair in patients with mitral stenosis after PBMV [14]. The results showed that the mean valve area using planimetry increased (1.16–1.62; *P* = 0.0001), the pressure half time using Doppler ultrasound decreased (202.4–152; *P* = 0.0001), and the mean pressure gradient using Doppler ultrasound decreased (9.4–5.8; *P* = 0.0021).

Based on these results, mitral valve repair can be suggested as an alternative method for patients with mitral restenosis who have received PBMV [14–19]. Song et al. (2010) found that patients with abnormal echocardiography findings and high atrial fibrillation rhythm showed better outcomes after MVS. This statement was confirmed by other studies [20–25], showing that MVS has advantages over PBMV because, in cases of the combination of MS with tricuspid regurgitation and atrial fibrillation rhythm, MVS is the best choice for treatment, but our study calculated atrial fibrillation rhythm as a possible confounding factor and found that it was not statistically significant to this research.

### Limitations

This study was a single-centre study using an observational study design with inherent limitations. Different follow-up times were unavoidable, but the median time follow-up in this study showed no significant differences between the groups. This study is too limited by the short follow-up period to determine survival in the PBMV and MVS groups. A longer time to follow-up and a larger sample size may have led to more reliable results.

## Conclusions

In this retrospective, observational single-centre study, the short-term safety and efficacy of PBMV was reconfirmed, as PBMV proved to be non-inferior to MVS in survival prognosis, but the sustained survival duration was significantly better in the MVS group.
